# Internet of Things (IoT) for Soil Moisture Tensiometer Automation

**DOI:** 10.3390/mi14020263

**Published:** 2023-01-19

**Authors:** Ahmed Ali Abdelmoneim, Roula Khadra, Bilal Derardja, Giovanna Dragonetti

**Affiliations:** Sustainable Water and Land Management in Agriculture, CIHEAM Bari, 70010 Valenzano, Bari, Italy

**Keywords:** precision agriculture, microcontroller, IoT irrigation, ESP32, sensors, BMP180

## Abstract

Monitoring of water retention behavior in soils is an essential process to schedule irrigation. To this end, soil moisture tensiometers usually equipped with mechanical manometers provide an easy and cost-effective monitoring of tension in unsaturated soils. Yet, periodic manual monitoring of many devices is a tedious task hindering the full exploitation of soil moisture tensiometers. This research develops and lab validates a low cost IoT soil moisture tensiometer. The IoT-prototype is capable of measuring tension up to −80 Kpa with R^2^ = 0.99 as compared to the same tensiometer equipped with a mechanical manometer. It uses an ESP32 MCU, BMP180 barometric sensor and an SD card module to upload the measured points to a cloud service platform and establishes an online soil water potential curve. Moreover, it stores the reading on a micro-SD card as txt file. Being relatively cheap (76 USD) the prototype allows for more extensive measurements and, thus, for several potential applications such as soil water matric potential mapping, precision irrigation, and smart irrigation scheduling. In terms of energy, the prototype is totally autonomous, using a 2400 mAh Li-ion battery and a solar panel for charging, knowing that it uses deep sleep feature and sends three data points to the cloud each 6 h.

## 1. Introduction

Water scarcity, exacerbated by climate change, is becoming a fast-spreading threat impacting livelihood, food security, economic development, and social stability. Globally, irrigated agriculture represents 70% of freshwater consumption [1,2], acting as both a major cause and a casualty of water scarcity. Thus, efforts towards enhancing on-farm irrigation management are crucial to face such challenges for the finite resource.

One of the main obstacles that hinders such efforts is the lack of cost effective and reliable data monitoring systems. Putting into consideration the spatially variable nature of agricultural systems, the availability of low-cost energy autonomous data collection means is crucial; spatial features mapping being an essential step towards more precise planning (scheduling), monitoring, and evaluation of irrigation events [3]. In fact, a combination of monitoring and modelling techniques is needed to both understand spatial variability impacts and assess the accuracy of the models [4]. In this study, a low cost IoT soil moisture tensiometer is introduced and lab validated to enable soil water potential mapping, visualizing, and archiving in real time on a cloud service platform.

### 1.1. Soil Water Potential: A Key Parameter

Together with soil moisture content, soil water potential is key information to describe a soil’s moisture condition which has an essential importance in dictating engineering, agronomic, geological, ecological, biological, and hydrological characteristics of the soil mass [5].

It could be defined as the energy that is needed to be exerted by plant roots to draw water from the soil, or it could be viewed as the force exerted by the soil matrix to hold the water [6]. The potential energy of soil water could be represented by Equation (1):

(1) $$\psi =\psi_{m}+\psi_{o}+\psi_{p}+\psi_{g}$$

where ψ is the potential energy per unit mass, volume, or weight of water and the subscripts *m*, *o*, *p*, and *g* are the matric, osmotic, pressure, and gravitational potentials, respectively [7].

Irrigation scheduling based on soil water potential—obtained usually by tensiometers—is a profound practical solution for rational use of water in irrigated agriculture [8]. Thompson et al. [9] define the soil water potential threshold for a specific crop-soil system as the threshold below which cultivated plants begin to suffer as a consequence of the decrease in the available soil water content. This eventually impacts the plants’ physiological well-being due to water stress [10]. Thus, identifying the appropriate soil water potential threshold for different crop-soil systems is an essential step towards more efficient irrigation management [6,9,10,11,12]

Soil water content must be coupled with the soil water potential readings to avoid over irrigation due to the inaccuracy of tensiometers under high tensions (80–100 kPa) especially in fine texture soils [5]. Other factors impacting soil water potential include soil texture, organic and inorganic content, and soil structure [6].

The applications of soil moisture tensiometers in irrigation management is well stated in the literature and several studies have discussed optimization of water productivity and irrigation scheduling based on recommended ranges of soil water potential for various crops [9,13,14,15].

### 1.2. Tensiometers: Advantages and Drawbacks of Widely Used Devices

Soil moisture tensiometers are one of the oldest and most widely adopted devices for measuring soil water tension, comprising a porous cup and a vacuum gauge for measuring the equivalent negative pressure or water tension in unsaturated soils [16]. Under saturation conditions, the reading on the vacuum gauge (usually a mechanical manometer) will be equal to the atmospheric pressure (pointing to zero). As soil moisture decreases, water is transferred from the tensiometer’s tube to the soil through the porous cup creating a negative pressure vacuum until equilibrium is reached. This negative pressure is detected by the manometer and represents the current soil water potential [17,18,19]. The porous cup wall should be permeable to water and have an air entry value greater than one atmospheric pressure even when it is saturated, to allow the air to escape once the soil moisture content is increasing again.

Since they are relatively cheap, easy to use, accurate, less prone to soil temperature and salinity, tensiometers are widely adopted for in-situ monitoring of soil moisture conditions [20,21,22]. However, one of the main drawbacks is water cavitation inside the tensiometer tube under high tensions (80–100 kPa) which can lead to inaccurate readings. Thus, periodical monitoring and maintenance are required to refill the tensiometer with de-aired water following cavitation [23,24,25,26]. Another important drawback is the small sensing area [2] which raises the need for multiple well-placed measuring points to account for the spatial variability caused by soil inhomogeneity, plant vigor, and/or low irrigation distribution uniformities, resulting from poor irrigation management/infrastructure. Dabach et al. [27] studied the tensiometer’s optimal placement in relation to the spatial variability of the soil hydraulic properties, root growth patterns, and plants’ root architecture under buried subsurface drip irrigation systems in sandy loam soil. The coefficient of variation (CV) for the tension ranged from 10% to 20% when the tensiometers were placed 10 and 20 cm from the drippers, while it was found to be lower (CV = 0–5%) when the tensiometers were placed at distances of 0 cm and 30 cm from the drippers.

As above-mentioned, the small sensing area coupled with vulnerability to cavitation has made regular systematic monitoring of a fleet of tensiometers scattered through the field a common practice, yet a tedious one. Thus, several efforts had been dedicated to automate soil moisture tensiometers. Thalheimer [19] developed a low-cost (72 euros) easy-to-build electronic soil moisture tensiometer using Arduino Uno microcontroller (MCU) and the piezoresistive differential pressure transducer MPX5100D (NXP simiconductors, Eindhoven, The Netherlands). The main objective was to reduce the costs and complexity of the prototype to facilitate rebuilding by potential users, thus data retrieval was done using the memory of the MCU board and collected by a mobile pc in situ every two weeks. Pereira et al. [28] built on the same concept using Arduino Mega MCU, pressure transducers, real-time clock, and an SD card module. The prototype was capable of measuring and recording the tension readings as a .txt file on the SD card coupled with a time stamp. It was then used to automate a drip irrigation system under various soil types using different tension thresholds according to each soil water retention curve. Sanches et al. [29] progressed the idea further by adding a DHT22 (AZDelivery—DHT2231—China) air temperature and moisture sensor to correct the fluctuations in the transducer readings due to the changes in the ambient temperature.

Although these efforts improved monitoring of soil water potential by automating periodical readings of the tensiometers along with data collection and storage in a cost-effective way, none found in the literature have considered developing an IoT-tensiometer capable of uploading the results into an easily accessible cloud service for real-time monitoring by farmers, operators, or researchers.

The aim of this work is to develop, and lab validate a low-cost, energy autonomous soil moisture tensiometer capable of measuring, recording, cloud storing, and visualizing soil water tension measurements in real time, on a web service platform.

## 2. IoT Tensiometer: Development of the Prototype

**Figure 1:**
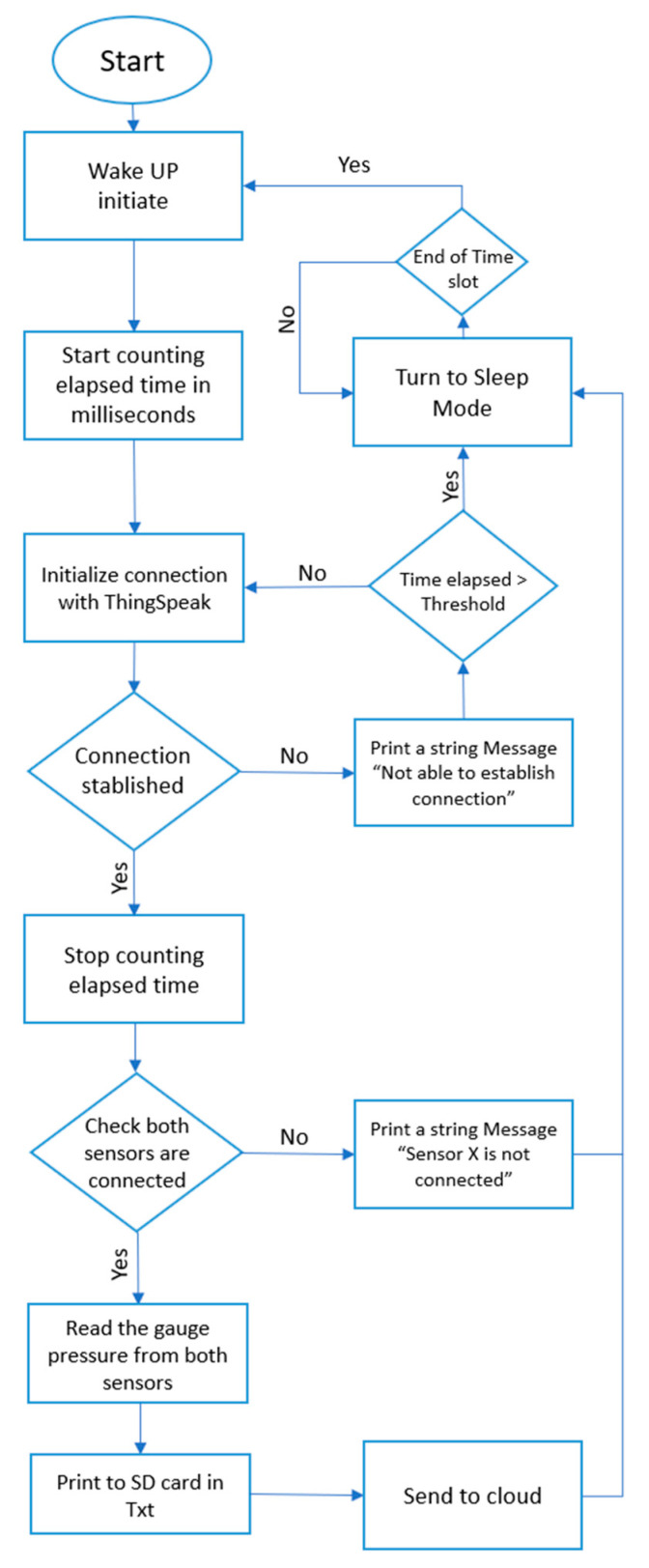
The algorithm flow chart.

The IoT-tensiometer uses an isolated BMP180 (ZHITING, Shenzhen, China) barometric pressure sensor placed at the top of the tensiometer tube just below the closing cap. The sensor is connected to the microcontroller (ESP32) by four thin wires (0.55 mm in diameter) using an inter-integrated circuit interface (I^2^C). The logic voltage for the BMP180 is 3.3 V and it can sense barometric pressure up to 110 kPa with high resolution (2 pa), making it ideal to sense any relative variation in the tensiometer vacuum. The brain of the prototype is the ESP32-WROOM-32D MCU (Espressif Systems, Shanghai, China) which is a low-cost powerful microcontroller module with an integrated WiFi and dual-mode Bluetooth. In this study, the deep sleep feature of the ESP32 was used to save energy consumption and guarantee the autonomous performance of the prototype. The ESP32 wakes up every 6 h (i.e., time slot is 6 h) to sense the tension in the tensiometer’s vacuum via the BMP180 sensors, write the data on the SD card, and send three points to the ThingSpeak cloud service, before going back into sleep mode. If the MCU does not find any available network for 30 s (i.e., “threshold” is 30 s), it prints the following string message as a .txt file on the SD card: “NOT able to establish connection”. It then goes back to sleep and wakes up again after 6 h. The same happens if the MCU could establish the connection but could not find the sensors, but the string message printed as .txt file indicates in this case: “Sensor x is not connected”. Figure 1 illustrates the code algorithm written in C++ language using Arduino IDE environment.

**Figure 2:**
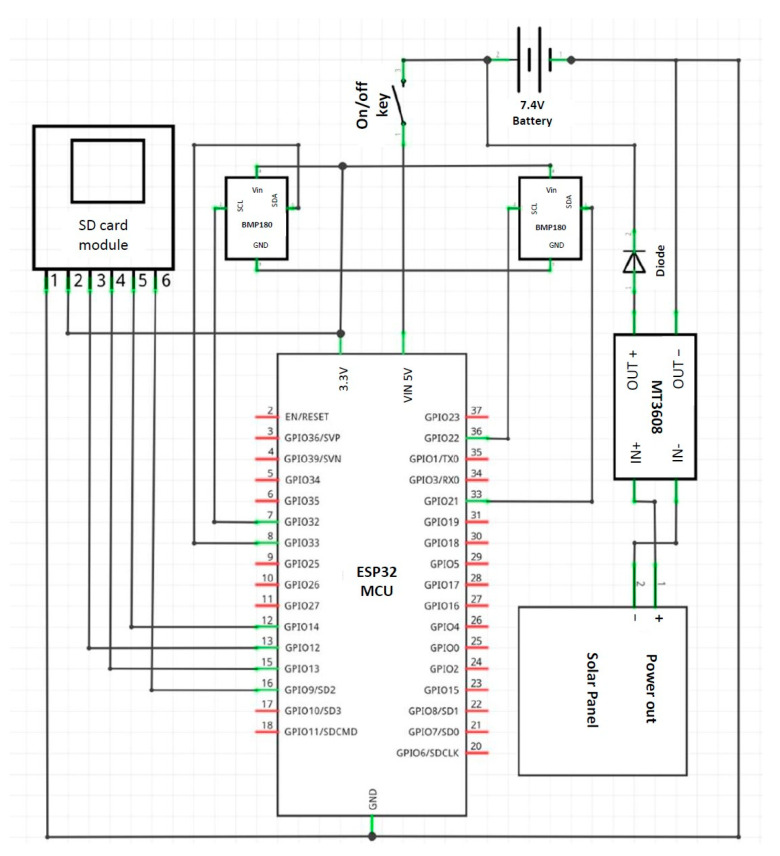
Connection scheme done with Fritzing.

**Figure 3:**
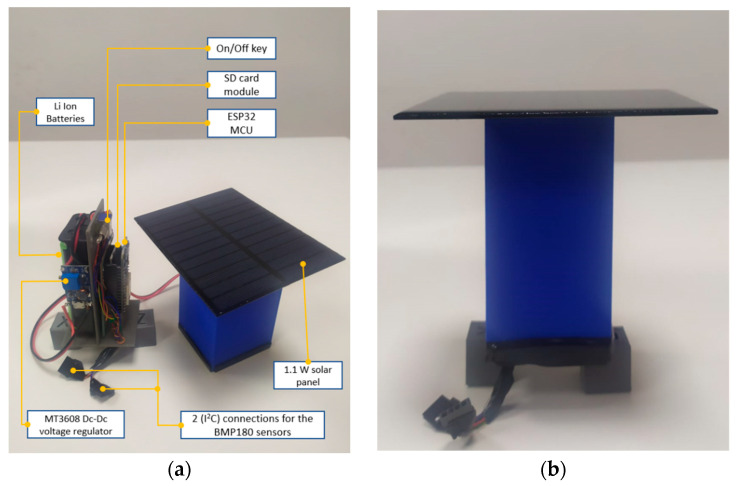
The IoT tensiometer prototype: (**a**) The components; (**b**) sealed prototype.

The prototype is powered by two Li-ion batteries, 3.7 V each and connected in series, thus giving 7.4 V. The batteries are being charged by a 1.1W solar panel through a MT3608 DC–DC voltage regulator to stabilize the input charging voltage coming from the panels to 9 V. MT3608 is a small-sized, low-cost step-up booster converter module, built for converting or boosting voltage as low as 2 V up to 28 V DC. As the ESP32 has two I2C connections, two sensors could be connected to each MCU, thus two tensiometers could be connected to each MCU Node. Figure 2 shows the connections scheme done with Fritzing software. All the components were mounted on a designed and 3D-printed platform using polyethylene terephthalate glycol material (PTEG). The design considered outdoor working conditions by minimizing any openings or holes, and by inserting the mounted electronic components as a core in a longer sleeve-like box with two tight side ducts (as a drawer) topped by the solar panel as shown in Figure 3.

There are many variants of microcontrollers (MCUs) from different manufacturers operating on the principle “Open-Hardware Platform”. In this study, the ESP32 was selected for being low cost, reliable, and compatible with the Arduino IDE programming environment [29]. It is used for multiple applications ranging from low-power sensor networks to the most demanding tasks. At the core of this module is the ESP32-D0WDQ6 chip with a dual core or single core LX6 microprocessor (Espressif Systems, Shanghai, China) that operates within the voltage range of 2.2 to 3.6 V. It has a 448 Kbyte Data ROM and 512 Kbyte Data SRAM [30]. Engineered for mobile devices, wearable electronics, and IoT applications, ESP32 achieves ultra-low power consumption through power saving features including fine resolution clock gating, multiple power modes, and dynamic power scaling.

The Internet of Things (IoT) refers to uniquely identifiable smart devices/objects connected to the internet that can sense data, react with their environment, and send the data into a web platform. Coupled with cloud computing, IoT is the driving engine of artificial intelligence (AI) and robotics into farming, both in large commercial and consumer level scales [30]. García et al. [31] did an extensive review of the IoT systems prototyping for irrigation in precision agriculture both as hardware and service web platforms. Eighty per cent of 160 studies were performed in the three years preceding this study (2017–2018–2019). The authors also found that ThingSpeak was the most identified service web platform used in research studies, while Arduino Uno MCU was the most utilized prototyping microcontroller especially for applications that do not require wireless sensor networks (WSN).

ThingSpeak™ (The MathWorks, Inc., Natick, MA, USA) is an IoT analytics platform service that allows for aggregating, visualizing, and analyzing live data streams in the cloud [32]. In this study, the platform was used as a tool for visualizing and publishing the collected data. The “internet thing” (in this case the prototype) connects to the assigned “field” through an application programming interface (API) that is referenced in the uploaded code on the MCU. The web platform (ThingSpeak) provides a time series data base, thus the received data for each field are plotted in real time. ThingSpeak is often used for prototyping and proof of concept IoT systems that require analytics.

## 3. Lab Validation of the IoT Tensiometer Prototype

**Figure 4:**
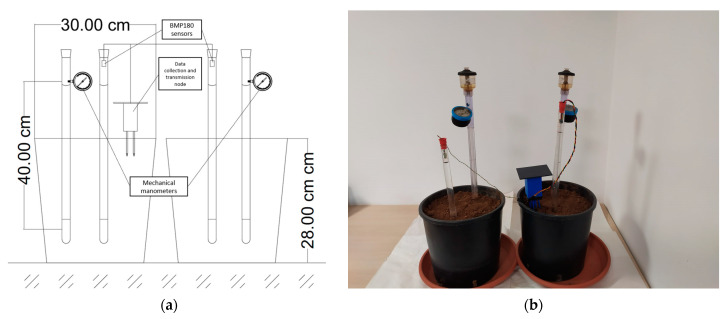
Validation setup: (**a**) Designed sketch; (**b**) in the lab.

A setup for the prototype validation was prepared in CIHEAM Bari- Italy soil lab as shown in Figure 4b, using a commercial tensiometer (JET FILL 2725, Santa Barbara, CA, USA) equipped with a mechanical gauge. The developed IoT tensiometer was assembled using a plexiglass tube 2 cm in diameter and 60 cm in length, a porous ceramic cup, and a rubber air-tight cap. An ultra-fine pin (0.5 mm) was used to insert four thin wires from the upper cap to connect the BMP180 pins: ground (GND), 3.3 voltage (VCC), serial data (SDA), and serial clock (SCL), then the top was sealed using isolation tap. The BMP180 sensor was fixed on the other side of the rubber cap. Both tensiometers were installed in a loamy silt soil. The dimensions for both pots used in the experiment are illustrated in Figure 4a. For error estimation, the validation was done simultaneously using two setups, thus four IoT tensiometers were validated.

As each developed node could be connected to two sensors, just one node was required for each validation setup to collect and send the data from the two IoT tensiometers. The readings from both tensiometers were registered daily: manually from the mechanical manometers, and automatically received on the cloud service or SD card in the case of the IoT prototype. The experiment started at soil saturation and stopped when −80 kPa tension was reached, as this is the operating range of a soil moisture tensiometer.

## 4. Results

**Figure 5:**
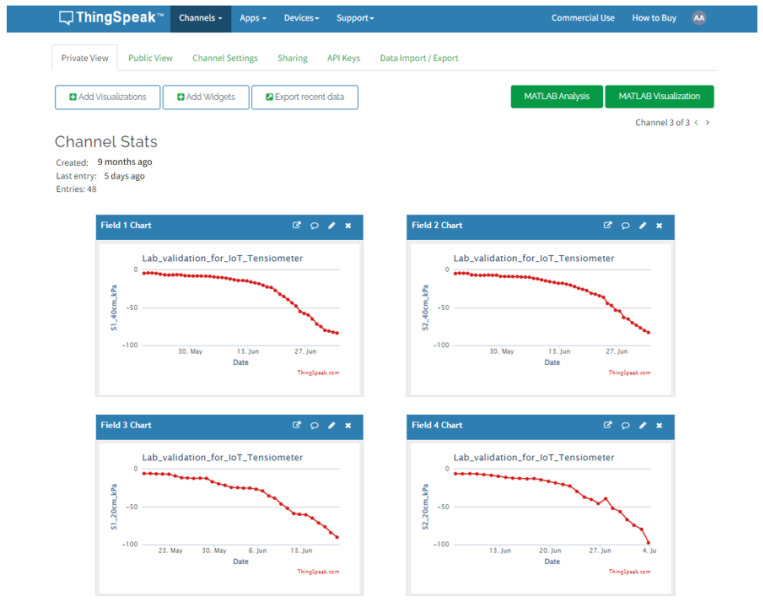
Online soil water potential curve from ThingSpeak.

The data, downloadable as an CSV, were received periodically (at least daily) over the dedicated ThingSpeak field (Figure 5). The deep sleep feature made the prototype fairly autonomous: it consumes less than one mA in sleep mode (0.8 mA) and 50 mA in 5 s, while uploading the data every 6 h. The solar panel charging current is around 100–120 mA on sunny days. Thus, a 2400 mAh Li-ion battery was more than sufficient to supply the prototype on dim cloudy days or at night during the test period (40 days from the end of May to the middle of July in south Italy).

**Figure 6:**
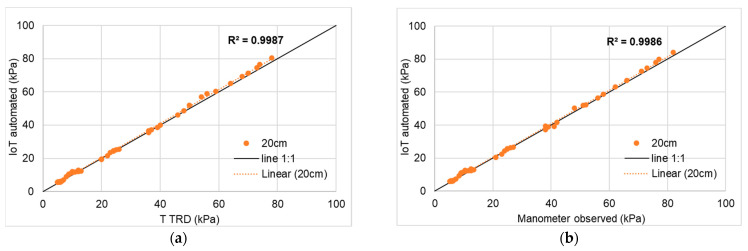

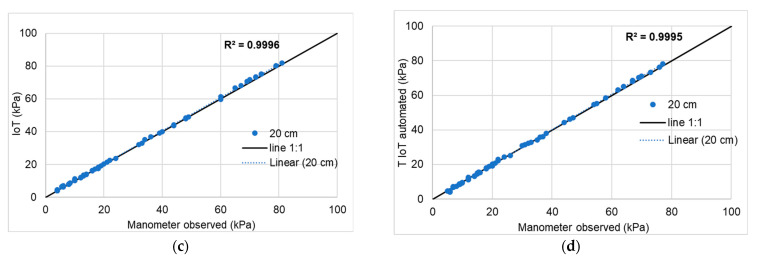
Comparison between the measured (observed) data and the automated feed: (**a**) and (**b**) First MCU node; (**c**,**d**) second MCU node.

The prototype was able to detect the tension in the whole expected operating range (from 0 to −80 kPa). As shown in Figure 6, the IoT tensiometer readings were almost identical to the one with mechanical gauge, under both soil depths with R^2^ = 0.99 in all four trials proving its overall reliability while the RMSE ranged between 0.7 to 1.1. This is mainly due to the variation in level of accuracy as the sensor can measure the tension with an accuracy of 2 pa while the minimum readable variation on the mechanical manometer dial is 1 kPa. Although previous studies achieved similar results using transducers where R^2^ = 0.99 [28,29], the added value of this prototype is its capability of uploading the results to a web platform in an energy independent cost-effective way.

The cost of the proposed prototype was relatively low (76 USD) as shown in Table 1, putting into consideration that the upgrade kit (the cap and the data collection node) could be attached to any matched tensiometer tube with a suitable diameter.

## 5. Conclusions

Advances in electronic technologies have provided researchers with access to low-cost, solid-state sensors, and programmable microcontroller-based circuits. Coupled with 3D printing potentials, prototyping for automating data collection has become much more feasible. In this study, an easy to assemble, cost effective, energy autonomous prototype for an IoT tensiometer was introduced and lab validated. The tensiometer is able to: measure the soil water potential with high accuracy (R^2^ = 0.99) up to −80 kPa; write the measured points on an SD card as a .txt file; and upload the data to a ThingSpeak field as a visualized online soil water potential curve which can be accessed using any mobile device (phone, laptop, tablet, etc.).

Such a prototype facilitates online soil water potential mapping in a cost-effective way and opens the door for more precise understanding of soil moisture spatial variability. Moreover, it supports studies on allowable water potential thresholds for various crop-soil systems, an essential parameter for better on-farm irrigation management. However, it is hindered by the reliability of the internet connection in remote areas, and thus could be further enhanced by using low-power wide-area network modulation techniques that do not depend on internet availability.

## Figures and Tables

**Table 1:** Breakdown cost of the prototype.

Item	Unit	Quantity	Cost $
ESP 32 WROOM	no	1	10
BMP 180 sensor	no	1	2.5
MT3608 DC–DC	no	1	2
SD card module	no	1	5
Tensiometer plexiglass tube	no	1	5
Permeable ceramic cup	no	1	4
2 cm airtight rubber cap	no	1	1
Li-ion batteries 3.7 volts	no	2	11
2S 18650 battery case	no	1	1
BMS 2S 10A charging model	no	1	4
1.1 W 6 V solar panel	no	1	14
Miscellaneous (wires, solder, isolation tape, pins, diodes, etc.)			15
PTGE filament	kg	0.5	1.5
Total			76

## Data Availability

Not applicable.
